# Prevalence and Distribution of Lesions in the Nasal Bones and Mandibles of a Sample of 144 Riding Horses

**DOI:** 10.3390/ani10091661

**Published:** 2020-09-16

**Authors:** Lucia Pérez-Manrique, Karina León-Pérez, Emmanuel Zamora-Sánchez, Sarah Davies, Christopher Ober, Bethany Wilson, Paul McGreevy

**Affiliations:** 1 Departamento de Etología, Fauna Silvestre y Animales de Laboratorio, Facultad de Medicina Veterinaria y Zootecnia, Universidad Nacional Autónoma de México, Av. Universidad 3000, Circuito Interior, Delegación Coyoacán, México D.F. 04510, Mexico; mvzlucia@gmail.com (L.P.-M.); nancy.karina.leon.perez@gmail.com (K.L.-P.); 2 Facultad de Medicina Veterinaria y Zootecnia, Universidad Autónoma del Estado de México, El Cerrillo Piedras Blancas, Toluca 50295, Estado de México, Mexico; emmanuel.zamorasan@gmail.com; 3 Veterinary Imaging Associates, 52-56 Atchison St, St Leonards, NSW 2065, Australia; daviesacvr@gmail.com; 4 Department of Veterinary Clinical Sciences, College of Veterinary Medicine, University of Minnesota, 1365 Gortner Avenue, St. Paul, MN 55108, USA; cpober@umn.edu; 5 Sydney School of Veterinary Science, Faculty of Science, University of Sydney, Sydney, NSW 2006, Australia; bethany.wilson@sydney.edu.au

**Keywords:** equitation science, welfare, radiology, nosebands, lesion

## Abstract

**Simple Summary:**

The use of restrictive nosebands in equestrian sports is of increasing concern to veterinarians and equitation scientists. Tightly fitting (restrictive) nosebands are primarily used to keep the horse’s mouth closed in a bid to increase the rider’s control of the horse and avoid penalties that may arise from mouth opening during competitions. The chief concern is that restricting behaviour by tightening the noseband may cause distress and apply pressure to the tissues of the horse’s head. It has been suggested that this pressure may cause injury to the soft tissues of the face and possibly the underlying bones. This opportunistic study of mature cavalry horses (*n* = 144) was designed to explore relationships between visual and palpable damage to structures that underlie the nosebands of horses and any related bony changes in affected horses, as evidenced by radiography. For nasal bones, the radiologists reported bone deposition in at least 6.9% of the horses and bone thinning in at least 33.3% of the horses, respectively. By palpation, at least 82% of the horses had palpable bone deposition of the nasal bones and at least 32% had palpable bone thinning. For the lower jaw, the radiologists reported increased bone deposition in 18.8–32.6% of the horses but no bone thinning. By palpation, at least 30.67% of the horses had palpable bone deposition in the lower jaw and at least 10.4% had palpable bone thinning. These radiographic results suggest that bone thinning is more apparent in the nasal bones than in the lower jaw and that both palpable and radiographic bone deposition are more likely in the mandible than in the nasal bones. This is the first confirmation of bony lesions at the site typically subjected to pressure from restrictive nosebands. That said, we note that the current study provides no evidence of a causal link between any piece of gear or its putative tightness and the lesions in these anatomical locations. The causes of these palpable and radiographic changes at the site of nosebands merit further investigation because inadvertently damaging the bones of horses as part of equitation is difficult to justify on ethical grounds.

**Abstract:**

Restrictive nosebands are used in equestrian sports to hold the bit in place and reduce mouth-opening, a response that can attract penalties in some sports and is thought to reduce the rider’s control of the horse. Sustained pressure from such tightly fitted (restrictive) nosebands denies normal behaviour and thus, causes frustration and distress that can jeopardise horse welfare. It also may push the cheek against the molar teeth, compress soft tissues including blood vessels and nerves, and possibly induce chronic changes to underlying bone. This study of mature cavalry horses (*n* = 144) was designed to explore relationships between visual and palpable damage to structures that underlie the nosebands of horses and any related bony changes in those horses as evidenced by radiography. Working independently of each other, two researchers inspected the horses for visual changes and palpable changes before the horses were radiographed. The radiographs were assessed by a separate pair of veterinary radiologists, again working independently of each other. Among the current population of horses, 37.5% had one or more radiographic changes to the nasal bones according to both radiologists, and 13.8% had one or more radiographic changes to the mandible. For nasal bones, the two radiologists reported bone deposition in 6.9% and 8.3% of the horses and bone thinning in 33.3% and 56.9% of the horses, respectively. By palpation, they found that 82% and 84% of the horses had palpable bone deposition of the nasal bones and 32% and 33.4% had palpable bone thinning. For the mandibles, the radiologists reported increased bone deposition in 18.8% and 32.6% of the horses but no bone thinning. By palpation, the two examiners reported 30.6% and 32.7% of the horses had palpable bone deposition and 10.4% and 11.1% had palpable bone thinning. This is the first report of lesions to the mandible at this site and this article presents the first confirmation of bony lesions at the site typically subjected to pressure from restrictive nosebands. These results suggest that radiographic bone thinning is more apparent in the nasal bones of riding horses than in the mandible and that both palpable and radiographic bone deposition are more likely in the mandible than in the nasal bones. That said, we note that the current study provides no evidence of a causal link between any piece of gear or its putative tightness and the lesions in these anatomical locations. Further studies are needed to identify risk factors for these clusters of lesions. The inadvertent deformation of bones in the horse’s head for competitive advantage is difficult to justify on ethical grounds.

## 1. Introduction

Nosebands may be used in horse-riding for an aesthetic effect (so-called framing of the horse’s face) and to keep the horse’s mouth closed [1]. By doing so, they may stabilise the bit in a preferred position and prevent the horse attempting to avoid bit pressure by putting the tongue out of the mouth or over the bit [2]. These behaviours are sometimes called evasions because they reduce the rider’s control of the horse and even attract penalties in some disciplines, notably dressage. 

Historically, an informal guideline for gauging noseband tightness has been to check if two adult fingers can be fitted between horse’s nose and the noseband [3]. The origin of this standard is unknown, but it has been appearing in equestrian texts since 1956 [4], up until the present [5,6,7,8,9]. However, perhaps as the perceived competitive advantages that were anticipated from tight nosebands became more broadly recognised, manual checking alone was questioned because of its imprecision [10]. One solution was the use of a standardised taper gauge such as that produced by the International Society for Equitation Science (ISES, [11]).

A recent European study of 750 eventing, dressage, and performance hunter horses [12] assessed the prevalence of tight nosebands in various equestrian disciplines. It found that 7% had space to accommodate 0.5 of a finger, 23% had space for 1 finger, 19% for 1.5 fingers, and 44% wore nosebands that allowed no space between the noseband and the skin over the nasal plane. Only 7% of nosebands would have passed the historic test and allowed for the placement of at least 2 fingers. 

Concern has been expressed globally that nosebands in some equestrian disciplines, such as eventing, have become progressively tighter over the past two decades to the point that their current tightness may now compromise horse welfare [10,13]. There is evidence that vascular perfusion may be compromised distal to restrictive nosebands, as a result of a tourniquet effect [14]. In naïve horses, tight nosebands are associated with transient stress responses and a post inhibitory rebound of behaviours, such as yawning, lip-licking, and chewing, that they restrict [15]. It has been shown that excessive noseband tightness is associated with lip abrasions and/or blood at the commissures of the lips [16]. Indeed, it has been suggested that that horses may bear evidence of remodelling of the nasal bones as a result of pressure from nosebands [10].

A pilot study using archived radiographs of horses’ heads (*n* = 60) [17] revealed that little is known about what constitutes normal appearance of the nasal bones radiographically. The relationships among palpable, visual, and radiologically confirmed changes, including exostoses and concavities, are of particular importance to the discussion about restrictive tightening of nosebands, since the changes detectable without radiography may provide warning signs that encourage carers to loosen nosebands before bony changes develop. It is possible that that both exostoses and concavities may occur in the same horse at the same time as simultaneous forms of remodelling. For example, it is possible that the apparent concavity is the result of remodelling of the bone distally. It is also possible that exostoses may visually and palpably accentuate a neighbouring minor morphological depression. Therefore, when a concavity arises adjacent to an exostosis, it may appear larger than were it to arise alone.

This article describes a non-invasive study of cavalry horses to determine what radiographic changes, if any, arise in equine nasal bones and mandibles at the point of contact with restrictive nosebands and how these changes relate to any palpable or visible changes at these sites.

## 2. Materials and Methods 

### 2.1. Horses

Warmblood horses (*n* = 144) were randomly selected from approximately 700 horses: 17 females and 127 males, weighing 450 to 550 kg, ranging in age from 3 to 18 years, and determined to be healthy by routine clinical examination, were used for this study. The horses were all housed at the Equine High-Performance Center (CEAR) of the Mexican army, in México City, where they begin their working career and start being trained for dressage, show-jumping, and eventing. Some horses also participate in army parades. Non-ceremonial gear for each horse depends on its equestrian activities and individual requirements. Ceremonial gear for this unit generally involves a curb bit and a cavesson noseband. Noseband tightness is not routinely checked with any taper gauge or similar device. For radiography, all horses were sedated with Xylazine 10% (0.5 mg/kg IV) and placed in a stock.

### 2.2. Experimental Procedure

The study was performed following the guidance of the National Guide for the Production, Care and Use of Laboratory Animals, Mexico (Norma Oficial Mexicana NOM-062–200-1999).

#### 2.2.1. Physical Evaluation

Two senior veterinary medicine students, blinded to the purpose of the study, were trained by the first author to examine the head of all horses for the presence of lesions, pain on palpation, and the presence of white hairs on the nasal bone at the site of the noseband and the mandible at the sites of the noseband and curb chain. 

For both visual inspection and palpation of the nasal bones (at the site of the noseband) and the mandible, these examiners were required to score the horse as normal or suspicious concavity, confirmed concavity, suspicious exostoses, or confirmed exostoses. They were also asked to note the colour of the horses and the presence of white hairs and skin damage at the site of the noseband and at the site of the curb chain.

#### 2.2.2. Radiographic Procedure

Approximately one week later, lateral radiographs, at 70 cm of distance, were taken 2 cm rostral to the facial crest, using a portable X-ray unit (Porta 100 HF^®^, Job, Yokohama, Japan) with a medical image processing unit (Edge Air^®^, Osko Inc., Medley, FL, USA). The radiographic technique used was standardised at 1.6 mAs and 76 kVp. 

In light of a pilot study [17], to refine technique, positioning, and radiographic exposure, scrupulous attention was paid to radiographic technique, bearing in mind the need for radiographs to be completely lateral with obliquity eliminated to the fullest extent possible. In addition, the whole region of interest, from the naso-incisive notch to the rostral extremity of the facial crest, was within the field of collimation. 

Radiographs were then studied by two specialists in diagnostic imaging, who were blinded to the age, breed, and sex of the horses studied. They were evaluated for any evidence of bone remodelling, soft tissue thickness, and radiographic opacity at the sites of the noseband as it meets the nasal bones and mandible. For lateral views of the nasal bones and mandible, the specialists were asked to score (as either absent, unsure, present (mild), present (moderate), or present (severe)) the following attributes: bone deposition; bone lysis; changes in bone homogeneity; fracture(s); and swelling of surrounding soft tissue. They were also asked to score the degree of obliquity in each image (using two options: satisfactory and unsatisfactory).

### 2.3. Statistical Analysis 

Statistical analysis examined relationships among visual inspection, palpation, and radiographic scores for the two sites (nasal bones and mandibles).

Cohen’s kappa was used to evaluate agreement between the physical exam scorers and between the radiographic scorers. For the physical exam scorers, an unweighted kappa was used to avoid assuming a natural order for exostotic and concavic change. Because radiographic scoring separated these features and scored them ordinally, a square weighted kappa was used for these scores, allowing consideration of the magnitude of disagreement. Calculations were made using the ‘irr’ package [18] of R statistical and computing software [19]. Two-sided Fisher’s exact tests were performed using the statistics package [19] of R to assess independence between findings of different diagnostic methodologies. If appropriate, post hoc pairwise tests for nominal independence were then completed using the ‘rcompanion’ package [20] with the Benjamini–Hochberg method used to control the false discovery rate.

## 3. Results

A variety of physically detectable and radiographic lesions were found in this population in the areas of the nasal bones and mandibles where we would expect to encounter noseband pressure.

### 3.1. Nasal Bone Lesions

#### 3.1.1. Physical Examination (Visual and Palpation Examinations)

##### Visual Inspection of the Nasal Bones

Exostosis lesions were visually detected more commonly than concavity lesions in this population although, depending on the examiner, 34% or 38% of the horses were at least suspicious for both kinds of change (see Table 1).

Therefore, for nasal bones, if we eliminate the normal horses and those considered to have suspicious but not confirmed changes, 32% or 33.4% (depending on the examiner) had palpable bone thinning and 82–84% had palpable bone deposition. 

Assuming no natural order to the categories above, there was a percentage agreement in categorisation of 68.1%. Cohen’s kappa was calculated to exclude agreement through chance and agreement was found to be moderate (κ = 0.612) and significantly greater than zero (z = 16.5, *p* < 0.001).

##### Palpation of the Nasal Bones

Palpation resulted in more findings of exostosis. The proportion of horses assessed to be at least suspicious for both sorts of change was similar—36% or 38% depending on the examiner (see Table 1). Assuming no natural order to the categories above, there was a percentage agreement in categorisation of 75.0%. Cohen’s kappa was calculated to exclude agreement through chance and agreement was moderate (κ = 0.633) and significantly greater than zero (z = 12.8, *p* < 0.001).

##### Visual versus Palpation Scores for Nasal Bones

**Figure 1:**
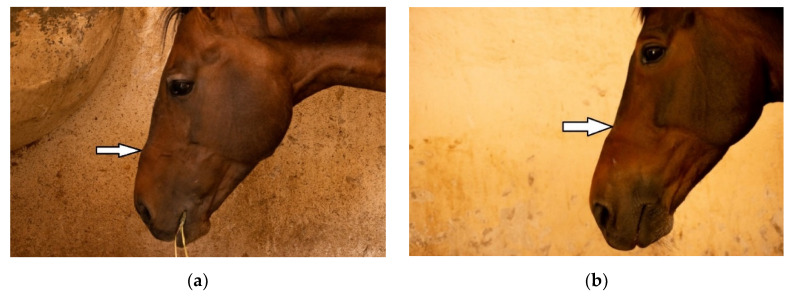
Photograph of profile of horse in which examiners (*n* = 2) agreed there was confirmed: (**a**) exostosis and (**b**) concavity in the nasal bones. Photographs courtesy of Missael García-Márquez.

Percentage agreement between visual and palpation characterisations were 41% for Examiner 1 (unweighted κ = 0.269, z = 7.72, *p* < 0.001) and were 43.8% for Examiner 2 (unweighted κ = 0.310, z = 8.89, *p* < 0.001). The most common difference in rating between the visual and palpation modalities for Examiner 1 (27 horses) was from suspicious exostosis visually (see Figure 1a) to confirmed exostosis by palpation. The most common difference in rating between the modalities for Examiner 2 (12 horses) was from suspicious concavity (see Figure 1b) and confirmed exostosis visually to confirmed exostosis and concavity by palpation. 

**Figure 2:**
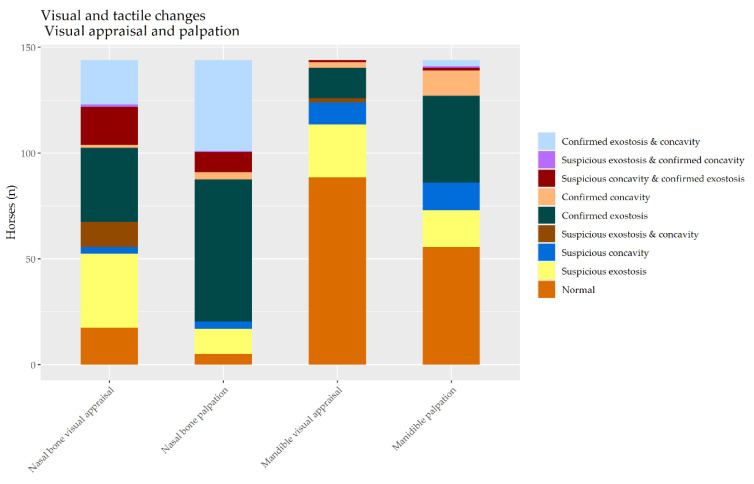
The distribution of visual and palpation findings in a random selection of 144 cavalry horses.

The distribution of the combined visual and palpation findings from the two examiners is shown in Figure 2.

##### White Hairs in the Nasal Bone Region

Of the 144 horses, 76 were assessed as having unnaturally white hairs in the nasal bone region, whilst 68 horses were judged as naturally white in the region due to markings or were grey. The examiners agreed that such hairs were present for 64.5% (*n* = 49 of 76) of the horses and absent for 30.3% (*n* = 23 of 76) of the horses. The remaining four horses were of disputed status. This led to a Cohen’s kappa of κ = 0.881 (z = 7.690, *p* < 0.001). The association between white hairs and a palpation finding in these 76 horses did not reach statistical significance by Fisher’s exact test (*p* = 0.11).

#### 3.1.2. Radiologic Examination

**Figure 3:**
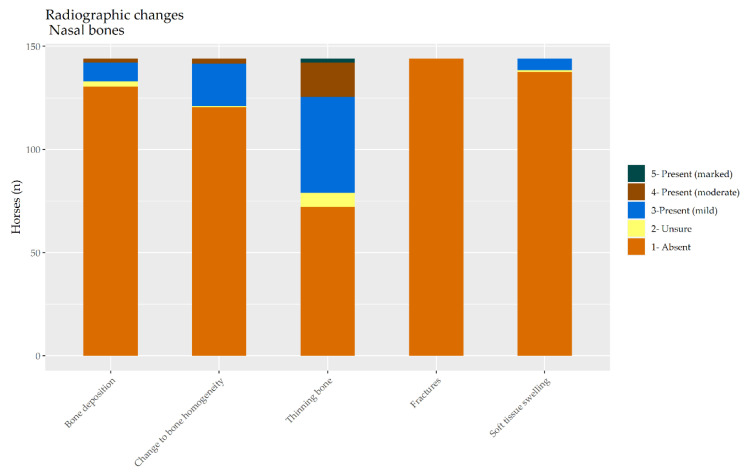
The distribution of radiological findings in the nasal bones of a random selection of 144 cavalry horses.

Among the 144 horses, 37.5% (*n* = 54) had one or more radiographic changes to the nasal bones according to both radiologists. Another 35.4% (*n* = 51) had no changes detected by either radiologist. For the remaining 39 horses, the radiologists disagreed about whether a lesion(s) was present. The distribution of radiological findings appears in Figure 3.

##### Horses with Consensus Change of Some Kind

**Figure 4:**
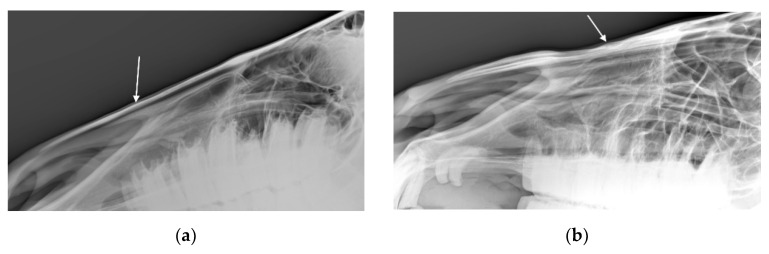
Radiographs showing the nasal bones of a horse in which radiologists (*n* = 2) agreed there was bone deposition that was: (**a**) typical of affected horses and (**b**) moderate.

**Figure 5:**
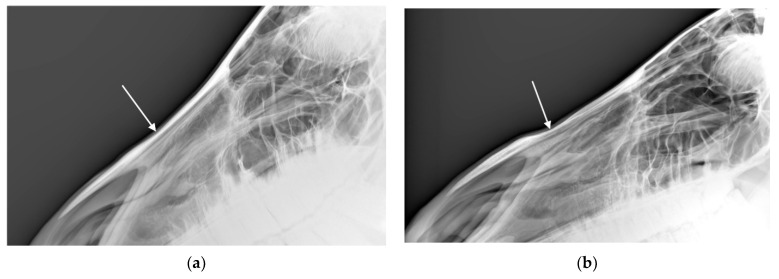
Radiographs showing the nasal bones of a horse in which radiologists (*n* = 2) agreed there was bone thinning that was: (**a**) typical of affected horses and (**b**) moderate.

For the 54 horses where the radiologists agreed there was at least one radiographic nasal bone change, the radiologists agreed about the presence or absence of bone deposition in *n* = 47 (87%; see Figure 4), the presence or absence of bone thinning in *n* = 45 (83%; see Figure 5), the presence or absence of loss of bone homogeneity in *n* = 43 (80%), and the presence or absence of soft tissue swelling in *n* = 49 (91%).

##### Horses with Disputed Change of Some Kind

For the 39 horses where the radiologists disagreed whether there was at least one radiographic nasal bone change, the radiologists agreed about the presence or absence of bone deposition in *n* = 35 (90%), the presence or absence of bone thinning in *n* = 4 (10%), the presence or absence of loss of bone homogeneity in *n* = 27 (70%), and the presence or absence of soft tissue swelling in *n* = 36 (92%). Disagreement about bone thinning was noticeably the most important source of dispute for these more equivocally affected horses (see Table 2).

##### Radiographic Assessor Agreement

(1) Bone Deposition

Bone deposition in the nasal bone region was noted on the radiographs of between 6.9% (*n* = 10) and 8.3% (*n* = 12) of the 144 horses. For most of these horses (*n* = 8–10), the noted change was mild. Marked changes were reported in no horses. Agreement was moderately good with a percentage agreement = 89.6% and a square weighted Cohen’s kappa = 0.622 (z = 7.47, *p* < 0.001)

(2) Bone Thinning

Bone thinning in the nasal bone region was more commonly noted than bone deposition. At least mild change was noted by one radiologist in 33.3% of the horses (*n* = 48 of 144) and by the other in 56.9% of the horses (*n* = 82 of 144). Most change was graded mild (*n* = 34–59), with some moderate (*n* = 13–20) and a few marked (*n* = 1–3). Agreement was fair but somewhat lower than for exostosis with a percentage agreement = 55.6% and a square weighted Cohen’s kappa = 0.494 (z = 6.88, *p* < 0.001)

(3) Loss of Bone Homogeneity

A radiographic finding of loss of bone homogeneity in the nasal bones was reported present more commonly than exostosis, but less frequently than bone thinning. Depending on the examiner, at least mild change was reported in 13.19% and 18.75% of the horses (*n* = 19–27 of 144). No horses were scored with marked change by either radiographer and moderate change was rare (*n* = 1–4 horses). Considering agreement between radiographers, while 81.9% of the horses were given the same score, the interpretation of the disputed horses was relatively varied, resulting in only a fair percentage agreement; square weighted Cohen’s kappa = 0.483 (z = 6.01, *p* < 0.001).

(4) Inflammation/Hypertrophy of Surrounding Soft Tissue

A radiographic finding of swelling of the soft tissue surrounding the nasal bones was the least commonly found of the nasal bone changes sought by this study, noted in only 5–6 of the 144 horses. Agreement was also relatively low, with a square weighted Cohen’s kappa of only 0.318, although this was still significantly higher than chance (z = 3.82, *p* < 0.001).

#### 3.1.3. Association between Physical Examination and Radiographic Examination Findings for Nasal Bones

##### Physical Examination Finding of Exostosis and Any Radiographic Lesion of the Nasal Bone

The relationship between a radiographic lesion and a physical exam finding for exostosis is shown in Table 3 below.

Palpation assessments were completed after the visual assessments. Therefore, one might expect that palpation could take visual assessment into account. The scores given by physical examiners for nasal bone exostosis were significantly associated with the finding of any radiographic lesions (Fisher’s exact test *p*-value = 0.005). Post hoc assessment showed that the physical examination findings were significantly different for the horses for which the radiologists agreed there were radiographic lesions and the horses for which the radiologists agreed there were no radiographic lesions (adjusted *p* = 0.013). Physical examination findings did not significantly differ for the horses in which radiologists disagreed on whether a radiographic lesion was present, either for the consensus normal (adjusted *p* = 0.096) or the consensus abnormal horses (adjusted *p* = 0.209).

When visual assessments (completed prior to palpation) were considered alone, the scores given by physical examiners were significantly associated with the finding of any radiographic lesion (Fisher’s exact test *p*-value = 0.007). In this case, post hoc testing showed no significant difference in visual findings for exostosis between horses with consensus radiographic change or disputed radiographic change (adjusted *p* = 0.875), but both these groups were significantly different from horses with consensus absence of radiographic change (adjusted *p* = 0.005 and 0.040, respectively). 

##### Physical Examination Finding of Concavity and Any Radiographic Lesion of the Nasal Bone

The relationship between a radiographic lesion and a physical exam finding for concavity is shown in Table 4 below.

Unlike the physical exam assessments of exostosis, there was no association between the presence of a radiographic lesion and the palpation of concavity on physical exam (*p* = 0.481) or the visual assessment of concavity on physical exam (*p* = 0.480).

### 3.2. Mandibular Lesions

#### 3.2.1. Physical Examination (Visual and Palpation Examinations)

##### Visual Inspection of the Mandible 

**Figure 6:**
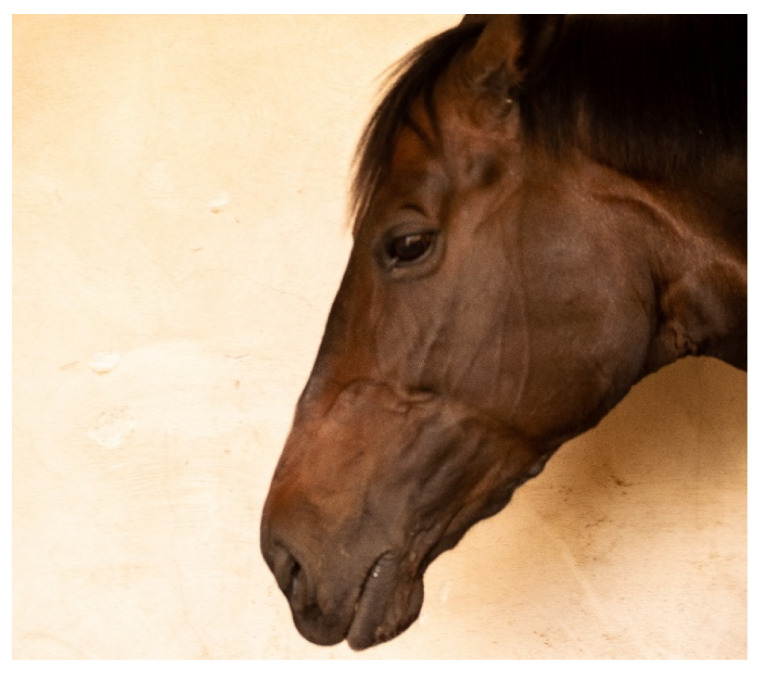
Photograph of profile of horse in which examiners (*n* = 2) agreed there was confirmed exostosis on the mandible. Photograph courtesy of Missael García-Márquez.

Exostosis lesions were visually detected more commonly (see Figure 6) than concavity lesions in this population and complex lesions visually showing both sorts of change are rare (up to 3.5% depending on examiner, see Table 5).

Therefore, for the mandibles, the two examiners reported that 10.4% and 11.1% of the horses had palpable bone thinning and 30.6% and 32.7% had palpable bone deposition. Assuming no natural order to the categories above, there was a percentage agreement in categorisation of 76.4%. Cohen’s kappa was calculated to exclude agreement through chance and agreement was found to be moderate (κ = 0.591) and significantly greater than zero (z = 11.6, *p* < 0.001).

##### Palpation of the Mandible

Palpation resulted in more findings of exostosis. The proportion of horses assessed to be at least suspicious for both sorts of change was a similar 3.5% for both examiners (see Table 5). 

Assuming no natural order to the categories above, there was a percentage agreement in categorisation of 71.5%. Cohen’s kappa was calculated to exclude agreement through chance and agreement was found to be moderate (κ = 0.591) and significantly greater than zero (z = 11.6, *p* < 0.001).

##### Visual Versus Palpation Scores for Mandible

Percentage agreement between visual and palpation characterisations was 51.4% for Examiner 1 (unweighted κ = 0.307, z = 7.10, *p* < 0.001) and 54.2% for Examiner 2 (unweighted κ = 0.354, z = 8.68, *p* < 0.001). The most common change in category for Examiner 1 (21 horses) was from suspicious exostosis visually to confirmed exostosis by palpation. The most common change in category for Examiner 2 (18 horses) was also from suspicious exostosis visually to confirmed exostosis by palpation. 

##### White Hairs in the Mandibular Region

Of the 144 horses, 127 were assessed for unnatural clusters of white hairs in pigmented skin in the mandibular region. Seventeen horses were judged as naturally white in the region due to markings or were grey. The examiners agreed that unnatural clusters of white hairs were present for 15.7% (*n* = 20 of 127) of the horses and absent for 77.2% (*n* = 98 of 127) of the horses. The remaining nine horses were of disputed status. This led to a Cohen’s kappa of κ = 0.773 (z = 8.73, *p* < 0.001). The association between white hairs and a palpation finding in these 127 horses was not significant by a Fisher’s exact test (*p* = 0.11).

#### 3.2.2. Radiologic Examination

**Figure 7:**
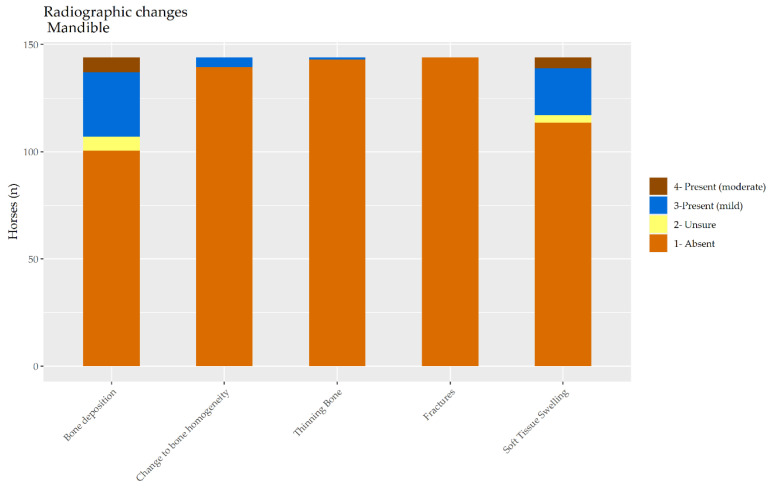
The distribution of radiological findings in the mandibles of a random selection of 144 cavalry horses.

Among the 144 horses, 13.8% (*n* = 32) had one or more radiographic changes to the mandible according to both radiologists. Another 63.9% (*n* = 9) had no changes detected by either radiologist. For the remaining 32 horses, the radiologists disagreed about whether a lesion(s) was present. The distribution of radiological findings in the mandibles is shown in Figure 7.

##### Horses with Consensus Change of Some Kind

**Figure 8:**
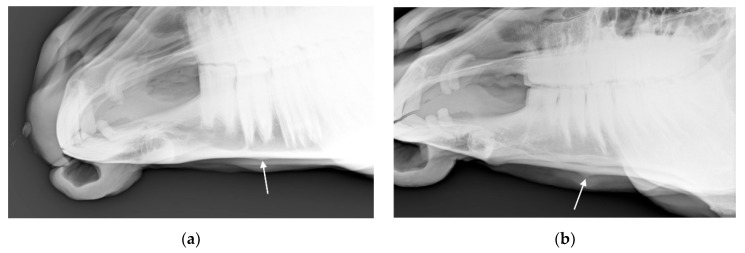
Radiograph showing the mandible of a horse in which radiologists (*n* = 2) agreed there was bone deposition that was: (**a**) typical of affected horses and (**b**) moderate.

**Figure 9:**
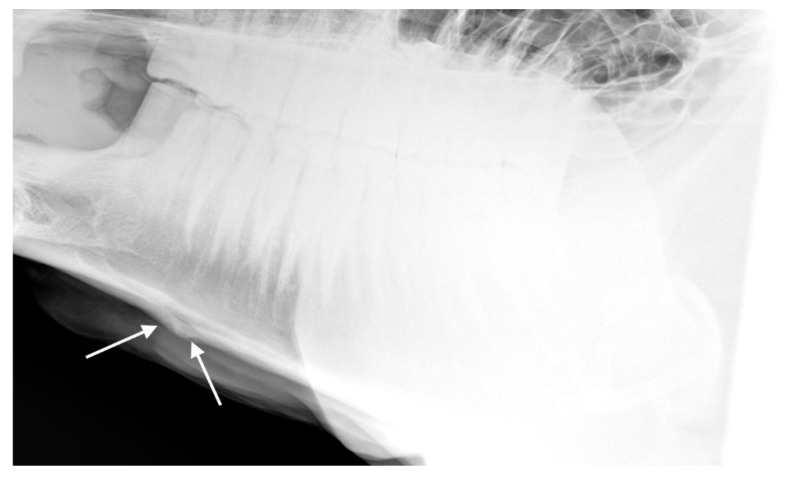
Radiograph showing the mandible of a horse in which radiologists (*n* = 2) agreed there was loss of mandibular homogeneity.

**Figure 10:**
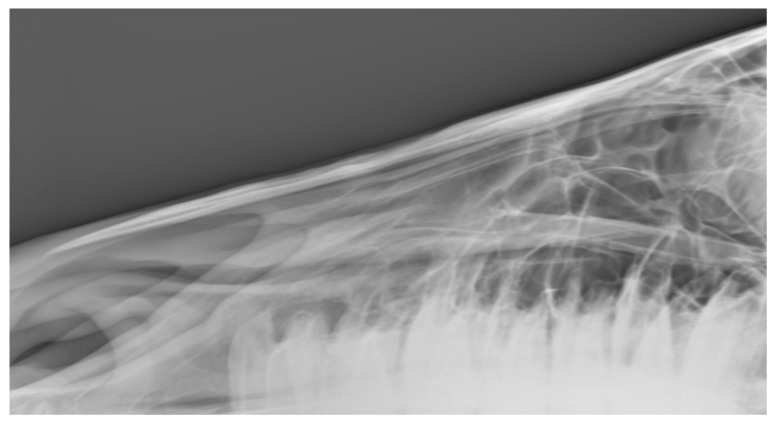
Radiograph showing the nasal bones of a horse in which radiologists (*n* = 2) agreed there was both bone thinning and bone deposition.

For the 32 horses where the radiologists agreed there was at least one radiographic mandible change, the radiologists agreed about the presence or absence of bone deposition in *n* = 22 (69%; see Figure 8), the presence or absence of loss of bone homogeneity in *n* = 25 (78%, see Figure 9), the presence or absence of bone thinning in *n* = 30 (94%), and the presence or absence of soft tissue swelling in *n* = 18 (56%). An example of a radiograph showing both bone thinning and bone deposition is shown in Figure 10.

##### Horses with Disputed Change of Some Kind

For the 20 horses where the radiologists disagreed whether there was at least one radiographic mandibular change, the radiologists agreed about the presence or absence of bone deposition in *n* = 2 (10%), the presence or absence of bone thinning in *n* = 20 (100%), the presence or absence of loss of bone homogeneity in *n* = 20 (100%), and the presence or absence of soft tissue swelling in *n* = 9 (45%). Disagreement about bone deposition especially and also soft tissue swelling was the source of dispute for these more equivocally affected horses (see Table 6).

##### Radiographic Assessor Agreement

(1) Bone Deposition

Bone deposition in the mandible was noted on the radiographs of between 18.8% (*n* = 27) and 32.6% (*n* = 47) of the 144 horses. For the majority of these horses (*n* = 19–41), the noted change was mild and changes in no horses were considered marked by either scorer. Agreement was moderately good with a percentage agreement = 79.9% and a square weighted Cohen’s kappa = 0.764 (z = 9.41, *p* < 0.001).

(2) Bone Thinning

Bone thinning in the mandible was noted only very rarely, in one horse per radiographer, a different horse in each case. For this lesion, agreement was not significantly different than chance square weighted Cohen’s kappa = −0.00699 (z = −0.0839, *p* = 0.933). 

(3) Loss of Bone Homogeneity

Loss of bone homogeneity in the mandible was also a rare finding, with only eight horses identified with mild change by either radiographer, with agreement only on one, leading to weak agreement over all; a square weighted Cohen’s kappa of 0.20 (z = 2.55, *p* = 0.011).

(4) Inflammation/Hypertrophy of Surrounding Soft Tissue

A radiographic finding of inflammation/hypertrophy of the soft tissue surrounding the mandible was frequent, although slightly less frequently than the most commonly found mandibular lesion of new bone deposition. At least mild change was noted in 14.6–22.9% of the horses (21–33%). Agreement was also relatively moderate, with a percentage agreement of 78.5% and a square weighted Cohen’s kappa = 0.59 (z = 7.37, *p* < 0.001).

#### 3.2.3. Association between Physical Examination and Radiographic Examination Findings for Mandibles

##### Physical Exam Finding of Exostosis and Any Radiographic Lesion of the Mandible

The relationship between a radiographic lesion and a physical exam finding for exostosis is shown in Table 7 below.

For palpation assessments, the scores given by physical examiners for mandibular exostosis were significantly associated with the finding of any radiographic lesion (Fisher’s exact test *p*-value < 0.001). Post hoc assessment showed that the physical exam findings were significantly different for the horses for which the radiologists agreed there were radiographic lesions, and the horses for which the radiologists agreed there were no radiographic lesions (adjusted *p* < 0.001). Physical examination findings of exostosis also significantly differed between the horses for which radiologists disagreed on whether a radiographic lesion was present and the horses for which there was a consensus of normality (adjusted *p* = 0.038) but did not differ between the horses for which there was a consensus of abnormality and the disputed abnormal horses (adjusted *p* = 0.078).

When visual assessments (completed prior to palpation) were considered alone, the scores given by physical examiners were significantly associated with the finding of any radiographic lesion (Fisher’s exact test *p*-value < 0.001). In this case, post hoc testing showed a significant difference in visual exam findings only between the consensus normal and the consensus abnormal horses (*p* < 0.001). The visual mandibular exostosis findings of the radiologically disputed horses did not differ significantly from either of the other groups. 

##### Physical Examination Finding of Concavity and Any Radiographic Lesion of the Mandible

The relationship between a radiographic lesion and a physical exam finding for concavity is shown in Table 8 below.

Unlike the palpation of exostosis, there was no association between the presence of a radiographic lesion and the palpation of concavity on physical exam (*p* = 0.779) or the visual assessment of concavity on physical exam (*p* = 0.614).

## 4. Discussion

We note that the current study provides no evidence of a causal link between any piece of gear or its putative tightness and the lesions in these anatomical locations. It is anticipated that the same cohort of horses will be studied to provide relevant data as we develop the design of a follow-up study. This will move from the current study, that is confined to prevalence and distribution at these anatomical locations, to risk factors for the various lesions reported here. It is expected that the follow-up study will explore the interactive role of multiple putative causal variables including: horse age; horse breed; horse sex; main equestrian disciplines (historic and current) as well as the 97 items of behavioural, management, and training data collected through the nascent Equine Behaviour Assessment Research Questionnaire (E-BARQ) instrument [21]. 

The risks of comprised welfare in horses wearing restrictive nosebands are widely recognised by equine welfare scientists [22]. The current opportunistic study is the first to reveal that physically detectable and radiographic lesions occur in areas of the nasal bones and mandibles likely to be subjected to pressure from restrictive nosebands. These findings are critical to the advance of ethical equitation [23] that advocates a three-step process for equestrian stakeholders who seek to retain the social license to operate [24]. It demands that we identify the causes of distress in the horses we ride, that we mitigate these stressors as much as possible, and we justify the retention of those that cannot be mitigated. 

A recent survey of horse owners revealed that 9% had observed swelling of the area under the noseband, 14.3% had observed soreness in the area under the noseband, and 39.9% had observed hair loss in the area under the noseband [25]. Of the subset of current horses suitably pigmented for assessment, 64.5% had white hairs in the nasal bone region, and 15.7% in the mandibular region. This suggests that the hairs of the nasal bones are more vulnerable than those over the mandibles to the type of injury that causes pigment changes. In both sites, the association between white hairs and a palpation finding was weak. Therefore, the presence of white hairs alone should be considered a poor indicator of lesions in these areas.

Among the current population, 37.5% had one or more radiographic changes to the nasal bones according to both radiologists, and 13.8% had one or more radiographic changes to the mandible. This indicates that the nasal bones are likely to be more vulnerable to changes. This may reflect the direction of the trauma that leads to these changes or the relative strength of mandibular bone. 

It is worth noting that when considering the nasal bones, exostosis was a more common finding on physical examination, yet, when examined by radiograph, bone thinning was generally more common than new bone deposition. Taken together with the finding that physical examination findings of exostosis were associated with one or more radiographic lesions in the nasal bone whereas physical examination findings of concavity were not, it may be that practitioners who believe they see or palpate exostosis on the nasal bones should have a higher index of suspicion for disease severe enough to cause radiographic lesions than those who believe they see or palpate concavity.

For both the nasal bones and mandibles, agreement on visual appraisal and palpation between observers was moderate and significantly greater than zero. We recommend that visual appraisal and palpation alone should not be relied upon diagnostically. As clinical indicators, they may well lead to imaging but there is evidence that radiological lesions are more profound.

For both the nasal bones and the mandibles, visual appraisal was more likely to detect increases in bone profiles than decreases. However, whereas at least 34% of the horses (depending on the examiner) were at least suspicious for both kinds of change in nasal bones, only up to 3.5% were suspicious for both kinds of change in the mandible. This suggests that observers are generally more familiar with normal appearance of the nasal bones than the mandible.

Palpation appears to be more sensitive to changes on both of these sites than visual appraisal, resulting in more findings of exostosis. Palpation confirmed exostosis in at least 45% of the nasal bones and 27% of the mandibles. Palpation confirmed concavity in at least 2.1% of the nasal bones and 8% of the mandibles. The proportion of horses assessed to be at least suspicious for both sorts of change was at least 36% but again depended on the examiner. 

Where there was agreement between the radiologists, 83% of agreement was about bone thinning in the nasal bones and 94% in the mandibles. In contrast, where there was disagreement between the radiologists about bone thinning, 10% of consensus was about bone thinning in the nasal bones and 100% in the mandibles. For bone deposition, where there was disagreement between the radiologists, 90% of consensus was about bone deposition in the nasal bones and 10% in the mandibles. Disagreement about bone thinning in the nasal bones was noticeably the most important source of dispute for these more equivocally affected horses. For the mandibles, disagreement about bone deposition and also soft tissue hypertrophy was the source of dispute for these more equivocally affected horses. This suggests that bone deposition is easier to detect with confidence in the nasal bones than in the mandible.

The limitations of the current study include its reliance on data from veterinary students who were not working in equine practice. That said, it is possible that seasoned equine practitioners may be inured to the presence of lesions at these sites in riding horses. Therefore, the two students who provided visual and palpation data for the current study may have benefited from approaching the question of normality without this possible habituation. 

The authors considered calculation of the negative predictive value (NPV) and positive predictive value (PPV) of the physical examination findings for radiographic change but ultimately, rejected them for two main reasons. Firstly, the NPV and PPV are affected by disease prevalence and the horses in the current study represent a unique population subject to unique equitation uses and husbandry that do not necessarily reflect the uses and husbandry of horses more broadly. Secondly, although radiography may be considered prima facie to be somehow ‘more objective’ than physical exam, it does not necessarily follow that radiology should be considered the ‘gold standard’ assessment tool for these lesions, and particularly, that horses who do not show radiographic disease should be considered unaffected by noseband injury. It is not uncommon, as a general principle for clinical signs to precede the development of radiographic change [26], and the lag between radiology and physical exam was up to several months, meaning that disease may have progressed between assessment by radiology and by physical exam.

Equine skulls are complex structures and so the current focus on the nasal bones meant that mA, kVp, exposure time, and focal spot–film distance were optimised for the nasal bones, not the mandibles. Future studies may benefit from treating the two sites separately. Longitudinal studies will be needed to reveal the role of various risk factors in the ontogeny of these lesions. There may be merit in separating the lesions of the mandible and nasal bones for such an exercise.

## 5. Conclusions

This article offers the first radiographic confirmation of bony lesions at the site typically subjected to pressure from restrictive nosebands and the first report of lesions to the mandible at this site. Among this population of military horses, 37.5% had one or more radiographic changes to the nasal bones according to both radiologists, and 13.8% had one or more radiographic changes to the mandible. These results suggest that radiographic bone thinning is more apparent in the nasal bones than in the mandible and that both palpable and radiographic bone deposition are more likely in the mandible than in the nasal bones. The identification of these lesions at the site of restrictive nosebands raises concern for the welfare of horses ridden with such devices. That said, we note that current study provides no evidence of a causal link between any piece of gear or its putative tightness and the lesions in these anatomical locations.

## Figures and Tables

**Table 1:** Distribution of categories of lesions in nasal bones (*n* and %) as detected by two examiners conducting visual inspection and palpation.

	Visual Inspection				Palpation			
	Examiner 1		Examiner 2		Examiner 1		Examiner 2	
	*n*	%	*n*	%	*n*	%	*n*	%
Normal	15	10.4	20	13.9	4	2.8	6	4.2
Exostosis								
Suspicious exostosis	37	25.7	33	22.9	13	9.0	11	7.6
Confirmed exostosis	37	25.7	33	22.9	69	47.9	65	45.1
Concavity								
Suspicious concavity	3	2.1	3	2.1	2	1.4	5	3.5
Confirmed concavity	3	2.1	0	0	4	2.8	3	2.1
Complex lesion								
Suspicious exostosis and concavity	12	8.3	12	8.3	0	0	0	0
Suspicious concavity and confirmed exostosis	18	12.5	18	12.5	10	6.9	9	6.3
Suspicious exostosis and confirmed concavity	1	0.7	1	0.7	0	0	1	0.7
Confirmed exostosis and concavity	18	12.5	24	16.7	42	29.2	44	30.6
Total	144		144		144		144	

**Table 2:** The average of the two radiologists’ scores as percentage of the number of horses affected. Types of radiographic lesion noted in those horses with consensus abnormal, disputed abnormal, and consensus normal radiographic films with respect to the nasal bone lesions included in this study.

Nasal Bone Radiographs	Consensus Abnormal	Disputed Abnormal	No Abnormality Detected
	*n* = 54	*n* = 39	*n* = 51
Bone deposition	19.4%	1.28%	-
Bone thinning	89.8%	42.31%	-
Loss of bone homogeneity	32.4%	14.10%	-
Soft tissue hypertrophy	8.3%	2.56%	-

**Table 3:** Relationship between any radiographic lesion being noted and the exostosis physical examination findings of the nasal bones of 144 horses.

	Radiologic Examination Outcome (Any Bone Deposition, Bone Thinning, Loss of Bone Homogeneity, or Surrounding Soft Tissue Inflammation/Hypertrophy of the Nasal Bone)					
	Consensus Disease		Disputed by Scorers		No Abnormality Detected	
**Exostosis present by palpation**						
	*n*	%	*n*	%	*n*	%
No suspicion	0	0	0	0	7	13.7
Suspicion from 1 of 2 scorers	1	1.9	1	2.6	3	5.9
Suspicion by both scorers	2	3.7	2	5.1	4	7.8
Confirmed by 1 scorer, no finding by 1 scorer	0	0	3	7.7	2	3.9
Confirmed by 1 scorer, suspected by 1 scorer	2	3.7	0	0	2	3.9
Confirmed by 2 scorers	49	90.7	33	84.6	33	64.7
**Exostosis present by visual examination only**						
No suspicion	2	3.7	1	2.6	12	23.5
Suspicion from 1 of 2 scorers	3	5.6	3	7.7	5	9.8
Suspicion by both scorers	10	18.5	10	25.6	16	31.4
Confirmed by 1 scorer, no finding by 1 scorer	1	1.9	1	2.6	1	2.0
Confirmed by 1 scorer, suspected by 1 scorer	5	9.3	5	12.8	3	5.9
Confirmed by 2 scorers	33	61.1	19	48.7	14	27.5

**Table 4:** Relationship between any radiographic lesion being noted and the concavity physical examination findings of the nasal bones of 144 horses.

	Radiologic Examination Outcome (Any Bone Deposition, Bone Thinning, Loss of Bone Homogeneity, or Surrounding Soft Tissue Swelling of the Nasal Bone)					
	Consensus Disease		Disputed by Scorers		No Abnormality Detected	
**Concavity present by palpation**						
	*n*	%	*n*	%	*n*	%
No suspicion	24	44.4	24	61.5	25	49.0
Suspicion from 1 of 2 scorers	4	7.4	0	0	4	7.8
Suspicion by both scorers	1	1.9	2	5.1	3	5.9
Confirmed by 1 scorer, no finding by 1 scorer	7	13.0	2	5.1	5	9.8
Confirmed by 1 scorer, suspected by 1 scorer	1	1.9	2	5.1	3	5.9
Confirmed by 2 scorers	17	31.5	9	23.1	11	21.6
**Concavity present by visual examination only**						
No suspicion	24	44.4	22	56.4	31	60.8
Suspicion from 1 of 2 scorers	6	11.1	3	7.7	7	13.7
Suspicion by both scorers	10	18.5	6	15.4	6	11.8
Confirmed by 1 scorer, no finding by 1 scorer	4	7.4	1	2.6	0	0.0
Confirmed by 1 scorer, suspected by 1 scorer	1	1.9	3	7.7	2	3.9
Confirmed by 2 scorers	9	16.7	4	10.3	5	9.8

**Table 5:** Distribution of categories of lesions in mandible bones as detected by two examiners conducting visual inspection and palpation.

	Visual Inspection				Palpation			
	Examiner 1		Examiner 2		Examiner 1		Examiner 2	
	*n*	%	*n*	%	*n*	%	*n*	%
Normal	86	59.7	91	63.2	57	39.6	54	37.5%
Exostosis								
Suspicious exostosis	28	19.4	22	15.3	15	10.4	20	13.9
Confirmed exostosis	17	11.8	12	8.3	43	29.9	39	27.1
Concavity								
Suspicious concavity	11	7.6	10	6.9	12	8.3	14	9.7
Confirmed concavity	1	0.7	4	2.8	12	8.3	12	8.3
Complex lesion								
Suspicious exostosis and concavity	1	0.7	3	2.1	0	0	0	0
Suspicious concavity and confirmed exostosis	0	0	2	1.4	1	0.7	2	1.4
Suspicious exostosis and confirmed concavity	0	0		0	1	0.7	0	0
Confirmed exostosis and concavity	0	0		0	3	2.1	3	2.1
					57	39.6	54	37.5
Total	144		144					

**Table 6:** The average of the two radiologists’ scores as percentage of the number of horses affected. Types of radiographic lesion noted in those horses with consensus abnormal, disputed abnormal, and consensus normal radiographic films with respect to the mandibular lesions included in this study.

Mandible	Consensus Abnormal	Disputed Abnormal	No Abnormality Detected
	*n* = 32	*n* = 20	*n* = 92
Bone deposition	87.5%	45.0%	-
Bone thinning	3.1%	0.0%	-
Loss of bone homogeneity	14.1%	0.0%	-
Soft tissue hypertrophy	68.8%	47.5%	-

**Table 7:** Relationship between any radiographic lesion being noted and the exostosis physical examination findings of the mandibles of 144 horses.

	Radiologic Examination Outcome (Any Bone Deposition, Bone Thinning, Loss of Bone Homogeneity, or Surrounding Soft Tissue Swelling of the Nasal Bone)					
	Consensus Disease		Disputed by Scorers		No Abnormality Detected	
**Exostosis present by palpation**						
	*n*	%	*n*	%	*n*	%
No suspicion	9	28.1%	7	35.0%	58	63.0%
Suspicion from 1 of 2 scorers	1	3.1%	3	15.0%	4	4.3%
Suspicion by both scorers	0	0.0%	0	0.0%	8	8.7%
Confirmed by 1 scorer, no finding by 1 scorer	1	3.1%	1	5.0%	3	3.3%
Conformed by 1 scorer, suspected by 1 scorer	1	3.1%	3	15.0%	8	8.7%
Confirmed by 2 scorers	20	62.5%	6	30.0%	11	12.0%
**Exostosis present by visual examination only**						
No suspicion	8	25.0%	12	60.0%	73	79.3%
Suspicion from 1 of 2 scorers	6	18.8%	2	10.0%	4	4.3%
Suspicion by both scorers	7	21.9%	4	20.0%	9	9.8%
Confirmed by 1 scorer, no finding by 1 scorer	2	6.3%	0	0.0%	3	3.3%
Conformed by 1 scorer, suspected by 1 scorer	1	3.1%	0	0.0%	1	1.1%
Confirmed by 2 scorers	8	25.0%	2	10.0%	2	2.2%

**Table 8:** Relationship between any radiographic lesion being noted and the concavity physical exam findings of the mandible of 144 horses.

	Radiologic Examination Outcome (Any Bone Deposition, Bone Thinning, Loss of Bone Homogeneity, or Surrounding Soft Tissue Swelling of the Nasal Bone)					
	Consensus Disease		Disputed by Scorers		No Abnormality Detected	
**Concavity present by palpation**						
	*n*	%	*n*	%	*n*	%
No suspicion	23	71.9%	13	65.0%	66	71.7%
Suspicion from 1 of 2 scorers	3	9.4%	3	15.0%	8	8.7%
Suspicion by both scorers	1	3.1%	1	5.0%	5	5.4%
Confirmed by 1 scorer, no finding by 1 scorer	1	3.1%	1	5.0%	8	8.7%
Conformed by 1 scorer, suspected by 1 scorer	1	3.1%	0	0.0%	0	0.0%
Confirmed by 2 scorers	3	9.4%	2	10.0%	5	5.4%
**Concavity present by visual examination only**						
No suspicion	26	81.3%	15	75.0%	79	85.9%
Suspicion from 1 of 2 scorers	4	12.5%	3	15.0%	7	7.6%
Suspicion by both scorers	2	6.3%	2	10.0%	2	2.2%
Confirmed by 1 scorer, no finding by 1 scorer	0	0.0%	0	0.0%	2	2.2%
Confirmed by 1 scorer, suspected by 1 scorer	0	0.0%	0	0.0%	1	1.1%
Confirmed by 2 scorers	0	0.0%	0	0.0%	1	1.1%
